# The Post of Matron

**Published:** 1918-11-09

**Authors:** 


### THE MATRONS' AND SISTERS' DEPARTMENT.

### THE POST OF MATRON.

### I.—How to Prepare for the Office.

To a probationer in the first year or two of her training the matron looms large as an awe-inspiring person, who must be obeyed, and whose decisions are final. She is regarded with envy and admiration as one who can "do just as she likes," give or withhold unlimited time off duty, or change a nurse from a ward in which she is "quite happy" to one where "Sister is so strict" or "the work is so dull." Matron has the right to find fault with the way the probationer's hair is done, the angle at which she puts on her cap, and all the hundred-and-one little shortcomings common to new probationers. "Oh, dear, how I wish I was a matron!" these victims say, and build a lovely castle, or rather hospital, in the air, where each sees herself the centre and sun of a happy and adoring staff; where the probationers are never found fault with, and all do just as they like, and are nevertheless the smartest and most perfect nurses, with the most beautiful and well-arranged wards, and the happiest and most contented patients. In this beautiful dream everybody chooses their own off-duty time, and arranges their holidays exactly as they wish, even if the staff all want the same month, and all nurses pass their final examinations with top marks from the matron.

But at the end of training, when the nurse has taken holiday charge of wards, she begins to have a dawning idea that a matron's work is not quite so easy and delightful as she fancied. Taking charge has opened her eyes to the meaning of responsibility, and to the necessity of forethought and patience, in order to keep the machinery of the ward running smoothly, and the patients properly attended to and happy. Then there are junior nurses to be looked after, and she finds out how much harder it is to see that work is done well by others than to do it oneself. When the nurse completes her training, and takes a sister's post, she comes into closer relations with the matron, and begins to understand the reason for arrangements which used to appear to her merely capricious, and to see that the matron is often obliged by circumstances to pursue a certain course, and is not really able to "do as she likes" at all.

At this point the sister sometimes doubts if she would really like to be a matron after all. At the completion of her training a nurse ought to have a general idea of what she wishes to aim at. Some do not look far ahead, and only wish for a post where the work is not hard, or where the salary is good, though there may be very little future prospect. There are others who are ambitious and desire to rise in their profession; and there are also many women who are really happiest in authority, although the work may be hard, the committee difficult to deal with, the nurses tiresome, and the patients' friends occasionally exacting and unreasonable. All nurses who aspire to become matrons should consider carefully whether they have reason to think that they possess the qualities and qualifications necessary for real success in such a position. The qualifications must be considered separately; but with regard to the qualities the following suggestions may be useful as a beginning:

Why do I want to become a matron? Is it simply because I like to be first, and to be at the head of something, or to be in a good position or have a large salary, or because I am just as good as Miss So-and-So, who is a matron? Or have I a different ideal altogether? Do I wish to be a friend to, as well as a trainer of, nurses, and to raise the standard of my profession in every way? Do I long to make hospital wards a real home for the patients by kindness, courtesy, and individual care for each sick person, whether his case is interesting or not? Have I good judgment, prompt decision, power of seeing both sides of a question, a strong sense of justice and fair play to everyone, whether I happen to like them or not? Can I uphold a high standard, and keep out of narrow grooves? Am I always ready to consider new ideas and methods, and not despise them because they are new? Do I really like responsibility, or am I anxious always to refer disagreeable decisions to someone else? Do I enjoy teaching, and can I speak plainly about mistakes and faults without hurting people's feelings? Can I always keep my temper, and speak courteously and gently, however rude or unpleasant other people are?

These are a few of the qualities necessary for a good matron—and nobody wants to be a poor one. Many sisters and assistant matrons always wish to refer anything that seems likely to be unpleasant to someone else. This may not matter much in a subordinate position, but a matron cannot avoid disagreeable interviews and unpopular decisions; and if she is constantly referring small matters to the medical superintendent or the committee, she will very soon be only a figurehead in her own hospital.

A would-be matron must prepare for her work also by taking trouble for others without expecting thanks, by learning to bear constant disappointments while never being disheartened, and to carry on with a cheerful face when things go as wrong as they can. These are some of the qualities of a good matron, though by no means all that are desirable. Her qualifications are a separate subject, equally important.

